# The Impact of COVID-19 Zoo Closures on Behavioural and Physiological Parameters of Welfare in Primates

**DOI:** 10.3390/ani12131622

**Published:** 2022-06-24

**Authors:** Ellen Williams, Anne Carter, Jessica Rendle, Sara Fontani, Naomi Davies Walsh, Sarah Armstrong, Sarah Hickman, Stefano Vaglio, Samantha J. Ward

**Affiliations:** 1 School of Animal, Rural & Environmental Sciences, Brackenhurst Campus, Nottingham Trent University, Southwell NG25 0QF, Nottinghamshire, UK; anne.carter@ntu.ac.uk (A.C.); samantha.ward@ntu.ac.uk (S.J.W.); 2 School of Veterinary Medicine and Science, Sutton Bonington Campus, Loughborough LE12 5RD, Leicestershire, UK; j.rendle@murdoch.edu.au; 3 Conservation Medicine, College of Science Health, Education and Engineering, Murdoch University, Perth 6150, Australia; 4 Twycross Zoo, Atherstone CV9 3PX, Warwickshire, UK; 5 School of Sciences, University of Wolverhampton, Wolverhampton WV1 1LY, West Midlands, UK; s.fontani@wlv.ac.uk (S.F.); sarah.ellen.hickman@gmail.com (S.H.); s.vaglio@wlv.ac.uk (S.V.); 6 Knowsley Safari, Prescot L34 4AN, Merseyside, UK; n.davies@knowsley.com (N.D.W.); s.armstrong@knowsley.com (S.A.); 7 Behaviour, Ecology and Evolution Research (BEER) Centre, Durham University, Durham DH1 3LE, County Durham, UK

**Keywords:** primates, visitor effect, behaviour, welfare, zoo/safari park

## Abstract

**Simple Summary:**

Zoo visitors can have a positive, negative, or neutral impact on animals. Primates are cognitively very advanced and their interactions with human visitors are complex. The COVID-19 pandemic led to a prolonged absence of visitors in zoos. This enabled an opportunity to compare how primates behaved when the zoo was open to visitors as opposed to when it was closed. We studied four primate species housed in the UK: bonobos, chimpanzees, and western lowland gorillas held at Twycross Zoo and olive baboons held at Knowsley Safari. Bonobos and gorillas spent less time alone when facilities were open to the public. Gorillas also spent less time resting when the facility was open to the public. Chimpanzees ate more and engaged more with enrichment when the zoo was open to the public. Olive baboons performed less sexual and dominance behaviour and approached visitor cars more frequently when the safari park was opened to the public than the ranger’s vehicle during closure periods. The results suggest that the zoo closures had variable impacts on the primates and that the closure periods were neither “negative” or “positive” for all of the studied species. There are likely to be differences between individuals due to prior experiences. We recommend that future work seeks to understand the impact individual differences and animal environments have on animals’ experiences with visitors.

**Abstract:**

Primates are some of the most cognitively advanced species held in zoos, and their interactions with visitors are complex. The COVID-19 pandemic provided a unique opportunity to understand the impact of zoo visitors on animals, in comparison to “empty zoos”. This study sought to understand the impact of facility closures and subsequent reopenings on behavioural and physiological parameters of welfare in four primate species housed in the UK: bonobos *(Pan paniscus)* (*n* = 8), chimpanzees *(Pan troglodytes)* (*n* = 11), and western lowland gorillas *(Gorilla gorilla gorilla)* (*n* = 6) held at Twycross Zoo (TZ); and olive baboons *(Papio anubis)* (*n* = 192) held at Knowsley Safari (KS). Behavioural data were collected from April–September 2020 (KS) and November 2020–January 2021 (TZ). Faecal samples were collected during morning checks from October–November (TZ) and July–November 2020 (KS). Faecal glucocorticoid metabolites (FGMs) were measured using ELISA kits. Statistical analysis for behavioural observations was undertaken using general linear models. Enclosure usage was assessed using t-tests and Mann–Whitney U-tests as appropriate. Bonobos and gorillas spent less time alone when facilities were open to the public (*p* = 0.004, *p* = 0.02 respectively). Gorillas spent less time resting when the facility was open to the public (*p* = 0.04), and chimpanzees engaged in more feeding (*p* = 0.02) and engagement with enrichment (*p* = 0.03) when the zoo was open to the public than when it was closed. Olive baboons performed less sexual and dominance behaviour and approached visitor cars more frequently when the safari park was opened to the public than they did the ranger’s vehicle during closure periods. There were no significant changes in physiological parameters for any of the study species. The results suggest variable impacts of the zoo closures on zoo-housed primates. We recommend future work that seeks to understand the impact of individual-level differences on “visitor effects” and that differences between animal experiences in zoos and safari parks are further explored in a range of species.

## 1. Introduction

One of the principal aims of modern zoos is to ensure animal welfare is kept to an optimum standard, with evidence-based approaches taken towards animal husbandry and management [1]. Zoos should be providing animals with the opportunity to thrive, not just survive [2,3]. As different animal species have different needs, zoological facilities now pay closer attention to the individual needs of animals, providing appropriate environments and assessing requirements for individuals, including enrichment [4] and training [5,6]. The zoo exposes animals to a unique environment full of novel stimuli [7]. One frequently mentioned and well-researched, unpredictable stimulus is the zoo visitor. The importance of human–animal interactions and the impacts of the presence of zoo visitors (referred to as “the visitor effect”) and animal-keeping staff have been increasingly recognised and have been further highlighted by the incorporation of “human relationships” into the Five Domains Model for zoo animal welfare [8]. 

Pre-COVID-19, zoo visitor numbers ranged from hundreds to millions per year, depending on the zoo [9]. The range in visitor number and the potential impact this may have on the animals’ behaviour and welfare spurred research into this area, with the results outlining the complex and multifaceted nature of this topic [10]. The impacts of zoo visitors can vary between facilities, between enclosures within facilities [11], and even between individual animals [12,13,14]. There are many factors which can impact the valence of the experience of visitors near to animal enclosures, including zoo visitor behaviour, enclosure design, presence or absence of enrichment, past experiences of individuals, and individual rearing history [10,15,16,17]. 

The stimulation caused by zoo visitors has been classed as positive, negative, or neutral, with animals described as perceiving visitors to be enriching, stressful, or showing indifference to them [10]. The relationship between zoo primates and visitors has been recognised as being highly complex and it has been suggested that primates may be particularly sensitive to “the visitor effect” [18]. The close relationship between zoo-housed primates and humans has led to a strong interest in the impact of zoo visitors on primates, with a plethora of work on this topic, the summary of which highlights the complex responses of primates to their zoo visitors. Lion-tailed macaques (*Macaca silenus*) displayed both short- and long-term changes in behaviour and enclosure usage, with animals performing more stereotypies and using the enriched zones of their enclosures more frequently when visitors were present within the zoo [19]. Similarly, in western lowland gorillas (*Gorilla gorilla gorilla*), increased prevalence of anxiety-related behaviours and decreased enclosure usage were observed [20], and white-cheeked gibbons (*Hylobates leucogenys*), although they displayed no behavioural changes, positioned themselves further away from visitors when higher densities of visitors were present, with greater periods of time spent out of sight [21]. Other research has indicated more mixed results, with some studies highlighting primate indifference to visitors. Carder and Semple [15] reported variations in behavioural response to visitors in two groups of western lowland gorillas, with one showing no negative responses and the other showing self-scratching and vigilance towards visitors (which was controlled through the offering of environmental enrichment). In orangutans (*Pongo* spp.), visitor number did not impact behaviour but when visitors were closer to the enclosure, increased vigilance and decreased play was observed [17]. Conversely, during a 12-month study of western lowland gorillas and chimpanzees (*Pan troglodytes*), Bonnie et al. [22] reported no behavioural changes and no changes to enclosure use in relation to visitor density at their enclosure.

As with the majority of the “visitor effect” literature, the majority of work investigating the impacts of human visitors on non-human zoo primates has been undertaken during zoo opening hours, with comparisons made between differing visitor numbers or impacts of visitor behaviour on animals. As a result of the COVID-19 global pandemic, zoological facilities were forced to close and there was concern over how animals would cope both without the visitors as a source of enrichment, and how they would cope, after having habituated to quieter environments during the lockdown period, when zoos reopened and visitors returned [23]. Long-term facility closures provided an opportunity to capture data on animal behaviour during periods of time when there were “no visitors” rather than “not many”, as in previous publications pre-COVID-19. This unique opportunity enabled an enhanced understanding of the “visitor effect” on zoo primates.

Despite some limitations in terms of study designs, research undertaken during the COVID-19-pandemic-enforced zoo closures has brought a new perspective on the experiences of zoo animals. A mixed behavioural response has been observed across species: slender-tailed meerkats (*Suricata suricatta*) engaged in more alert behaviour and fewer positive social interactions during closures than open periods [24] and giraffe (*Giraffa camelopardalis tippelskirchi*) reduced vigilance when visitors returned to zoos following the closure period [25]. Chinese goral (*Nemorhaedus griseus*) engaged in more environmental interactions when the zoo was closed to the public, red kangaroos (*Macropus rufus*) increased inactivity, spent more time in proximity to one another, and reduced their space use when the zoo reopened [26], and amphibians (common toad, *Bufo bufo*; common frog, *Rana temporaria*; smooth newt, *Lissotriton vulgaris*; pool frog, *Pelophylax lessonae*; golden mantilla, *Mantella aurantiaca*; golden poison dart frog, *Phyllobates terribilis*) were less visible when zoo visitors returned [27]. Behavioural indifference was reported in Nile crocodiles (*Crocodylus niloticus*) [11] and flamingos (*Phoenicopterus chilensis*) when the zoos opened [28]. 

Enclosure design has been shown to influence the animals’ response to visitors [10]. In a typical zoo enclosure, animals are housed within, and visitors surround the enclosure with various viewing opportunities. However, at a safari park, drive-through enclosures are common. Here the animals are within large expansive enclosures and the visitors drive in vehicles along predetermined roads within the enclosures i.e., the visitors are “contained” within an enclosed space (vehicle) and animals are comparatively more free to choose to approach or avoid them. To the authors’ knowledge, no published research has investigated the implications, if any, of these changes in exhibit scenarios on animal wellbeing and behaviour. However, an unpublished study undertaken at Knowsley Safari indicated that camels (*Camelus bactrianus*) born in conventional zoo environments who were transferred to the safari park were significantly more likely to change their behaviour (from one activity to another) when visitors were present at the safari park. This was in comparison to the behaviour of their conspecifics who had been born within the safari park. The differences were believed to be related to the nature of human–animal interactions in the two settings [29]. Although the focus of that research was on impacts of rearing history, there is potential for differing responses to zoo visitors in the two differing environments.

The aim of the present research was to use the COVID-19 facility closures and subsequent absence of visitors to advance our understanding of the “visitor effect” in a selection of zoo primates with the use of behavioural and physiological parameters of welfare. We expected to see one of three characteristic responses to the return of zoo visitors when facilities were open to the public (in between periods of closure): excitement, stress, or indifference. Due to the repeated facility closures and subsequent reopenings, we hypothesised that there would be differences in animal behaviour and faecal glucocorticoid metabolites (FGMs) (at both study sites) and enclosure use (at Twycross Zoo) between periods when the zoo was open to the public and when it was closed to the public. We further hypothesized that olive baboons at Knowsley Safari would show changes in behaviour and FGMs in response to increased numbers of cars in the enclosure. 

## 2. Materials and Methods 

### 2.1. Subjects and Study Sites

Bonobos (*Pan paniscus*), chimpanzees, olive baboons (*Papio anubis*), and western lowland gorilla held at two zoological collections in the United Kingdom were studied. Facilities were closed from mid-March 2020 and reopened mid-June 2020 before closing again from the start of November to December 2020. Details of the demographics of the study individuals and periods of data collection are provided in Table 1. 

### 2.2. Behavioural Observations

Observation time periods were kept consistent within species and did not occur during periods when keepers were specifically interacting with the animals (e.g., during feeding times or training procedures). Due to differences in behavioural sampling protocols, the two sites are treated as separate entities and detailed methods are described below. Comparisons are made between sites in the discussion to draw overarching conclusions.

#### 2.2.1. Twycross Zoo—Behavioural Observations and Enclosure Usage

Behavioural observations were undertaken using instantaneous scan sampling with a 5 min inter-scan interval [30] for approximately six hours per day, for 9 to 10 days per species (Table 1). Scans of the enclosure were taken from left to right and the number of individuals performing each behaviour was recorded. Behaviours were recorded according to a predefined ethogram (Table 2). The enclosure usage was recorded at the same time as the behavioural data collection. Enclosures were visually split into five equal areas according to their proximity to the public viewing area (Zone 1: closest to visitor viewing area, through to Zone 5: furthest from the visitor viewing area). 

#### 2.2.2. Knowsley Safari

Data collected at Knowsley Safari (KS) were collected as part of routine long-term behavioural monitoring of the olive baboons. Behaviours were recorded according to a predefined ethogram (Table S1) and then consolidated into overarching behavioural types (Table 3). Data were collected once per day, 3–5 times per week, with the observation period lasting 30 min. Data were collected using instantaneous scan sampling with a one-minute inter-scan interval [30]. Behaviours were recorded as present or absent (within the focal group, i.e., regardless of how many animals performed the behaviour) at each behavioural scan of the focal group. Visitor numbers were recorded as the average number of cars (including keeper vehicles) per minute for the 30 min observation period. Rates of human–animal interactions per average number of cars (including keeper vehicles) were calculated to investigate whether interaction rates varied during open and closed conditions. 

### 2.3. Faecal Sampling

#### 2.3.1. Sample Collection

Faecal samples were collected regularly by animal keepers during open and closure periods (Table 4). At TZ, samples were collected during October and November 2020, while at KS, samples were collected from July to November 2020. Fresh samples were collected during normal husbandry routines between 08:00–11:00. Due to staffing constraints during the COVID-19 pandemic and the opportunistic study design, it was not possible to individually identify faecal samples for any of the study species. Single (unidentified) faecal samples were collected from chimpanzee, gorilla, and bonobo enclosures at TZ. Pooled samples were collected from baboons at KS. Samples were placed into an Eppendorf vial, labelled with details about sampling date and species, and then immediately stored at −18 °C on site. Samples were transported to the University of Wolverhampton in a freezer box containing additional freezer blocks to avoid any risk of defrosting. Samples were then placed in a −20 °C freezer prior to endocrinology analyses.

#### 2.3.2. Sample Preparation and Extraction

Faecal samples were lyophilized for 72 h using a freeze-drying machine (Christ^®^, Beta 1–8 LSC plus, Osterode am Harz, Germany) and pulverised using a pestle and mortar; the powder was sieved through a stainless-steel strainer to separate the faecal residue from the fibrous material. The extraction methodology was modified from the methods of Maréchal et al. [35] and Fontani et al. [36]. Briefly, 0.1 g of faecal powder was extracted in 3 mL of 80% methanol in a 15 mL plastic tube; after vortexing for 15 min using a multi-tube vortexer (Grant Instruments^®^, Multi-Vortexer V-32, Cambridge, UK) and centrifugation for 20 min at 3266× *g*, the supernatant was immediately stored at −20 °C.

#### 2.3.3. Enzyme Immunoassay

Cortisol metabolite levels were measured using a Cortisol ELISA kit (Enzo Life Sciences^®^, ADI-900-071, New York, NY, USA) that has broad cross-reactivity and has previously been validated for faecal samples assessment in other mammal species [37,38], including primates [39]. According to the manufacturer, the cross-reactivity was cortisol (100%); prednisolone (122.35%); corticosterone (27.68%); 11-deoxycortisol (4.0%); Progesterone (3.64%); prednisone (0.85%); testosterone (0.12%) and <0.10% with androstenedione, cortisone, and estradiol. The sensitivity of the assay was 56.72 pg/mL (range 156–10,000 pg/mL). Samples were diluted 1:10 with the assay buffer. All faecal samples and standards were assayed in duplicate. Assay data were analysed utilising a 4-parameter logistic (4PL) fitting programme (MyAssays^®^, Brighton, UK). Intra-assay coefficients of variation at low, medium, and high concentrations of cortisol were 10.5%, 6.6%, and 7.3%, respectively; inter-assay coefficients at low, medium, and high concentrations of cortisol were: 13.4%, 7.8%, and 8.6%, respectively.

#### 2.3.4. Analytical Validation

A parallelism test between serial dilutions of faecal extracts and the standard curves was conducted to validate the enzyme immunoassay [40]. One faecal sample per species was diluted (1:10 to 1:160) using an assay buffer. Diluted samples were then assayed together with cortisol standard (serial dilution 10,000–156 pg/mL). A Spearman rank correlation test was performed to assess the strength of the association between the slope of the standard curve and the slopes of the diluted samples (r_s_ = 1, *p* = 0.01).

### 2.4. Ethics Statement

All research protocols were approved by Nottingham Trent University, School of Animal, Rural and Environmental Sciences School Ethics Group (reference number ARE192042) and meet the ARRIVE guidelines where necessary. Permission to conduct the study was granted by the participating zoos prior to commencement of data collection. 

### 2.5. Data Analysis

#### 2.5.1. Behavioural and Enclosure Use Data

Negative binomial general linear models (GLMs) were used to investigate the relationship between observed behaviour and data collection period (facility closed vs. facility open to the public). Number of observations of each behaviour per observation period was fitted as a response variable in each model. In all models, data collection period (open to the public/closed to the public) was fitted as a fixed effect. For bonobos, chimpanzees, and gorillas, the total number of observations during each observation period was fitted as an offset variable in order to control for slight variation in the length of observation periods. In baboons, the focal group size category (see Table 5 for group size categories) was fitted as an offset variable to account for variability in the size of the group being observed. 

Separate models were created for each species. Analyses were undertaken using R (Version 4.0.3) [41] using package “MASS” [42]. Model results are reported as model estimate (β_1_) ± SE. Significance values were set at *p* < 0.05. Full model outputs for all models are reported in Table S2. Appropriateness of models was assessed by visual examination of dispersion of residuals. Final models were selected using AIC values.

Enclosure usage in the TZ primates was assessed by comparing the average number of individuals within each enclosure zone (Zones 1–5) per observation day during open and closed periods to determine whether number of individuals within each zone varied on open and closure days. Analysis was undertaken using a t-test for independent samples or a Mann–Whitney U-test, according to normality of the data and homogeneity of variance, using SPSS Version 26 (SPSS Inc., Chicago, IL, USA). Homogeneity of variance was assessed using a Levene’s test for all datasets. Where homogeneity of variance was not met (enclosure usage data: bonobos and chimpanzees Zone 5 and gorillas Zone 2), data were log-transformed using the transformation of ln(x) if there were no zeros in the dataset (gorilla and chimpanzees) and ln(x + 1^−10^) if zeros were present (bonobos).

An overview of behavioural analysis models created and statistical tests undertaken to assess enclosure usage across species is included in Table 6. 

#### 2.5.2. Faecal Glucocorticoid Metabolites

The relationship between amounts of FGMs and open/closed periods was undertaken using a Mann–Whitney U-test for baboons and bonobos. An unpaired t-test was used to assess FGMs during open and closed periods for chimpanzees and gorillas. A Levene’s test for equal variances was undertaken to determine whether there was greater variation in FGMs during open or closed periods. 

## 3. Results

### 3.1. Twycross Zoo

#### 3.1.1. Frequency of Behaviour and Enclosure Usage

**Figure 1:**
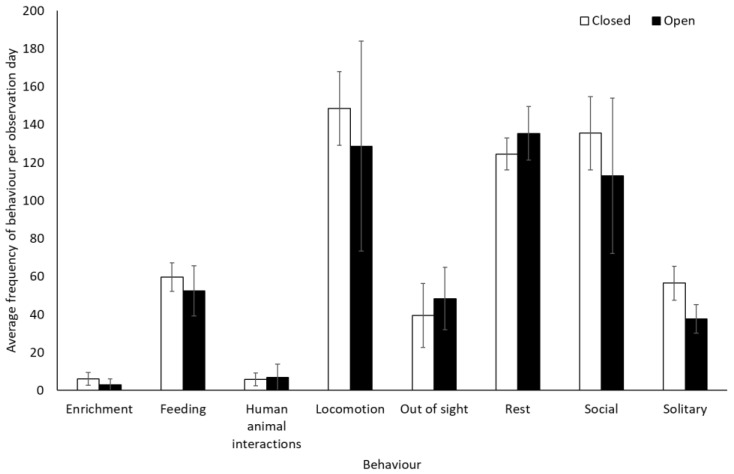
An overview of mean frequency of bonobo behaviour during closure and reopening observation periods. Error bars represent standard deviation.

**Figure 2:**
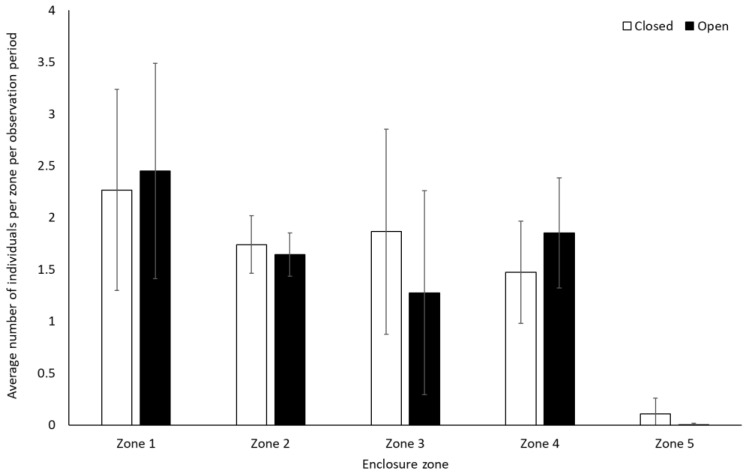
Average number of bonobos per enclosure zone per observation period. Error bars represent standard deviation.

Bonobos spent less time by themselves (solitary) when the zoo was open to the public compared to when it was closed to visitors (−0.312 ± 0.11, Z = −2.88, *p* = 0.004). There were no other significant differences in frequency of behaviour between periods when the zoo was open or closed to the public for the bonobos (*p* > 0.05, Figure 1, Table S2). They also showed no significant difference in use of the five enclosure zones between open and closure periods (Zone 1: t(8) = −0.28, *p* = 0.79; Zone 2: U = 7.00, df = 8, Z = −1.07, *p* = 0.35; Zone 3: U = 4.00, df = 8, Z = −1.71, *p* = 0.11; Zone 4: U = 6.00, df = 8, Z = −1.28, *p* = 0.26; Zone 5: U = 7.00, df = 8, Z = −1.31, *p* = 0.19) (Figure 2). 

**Figure 3:**
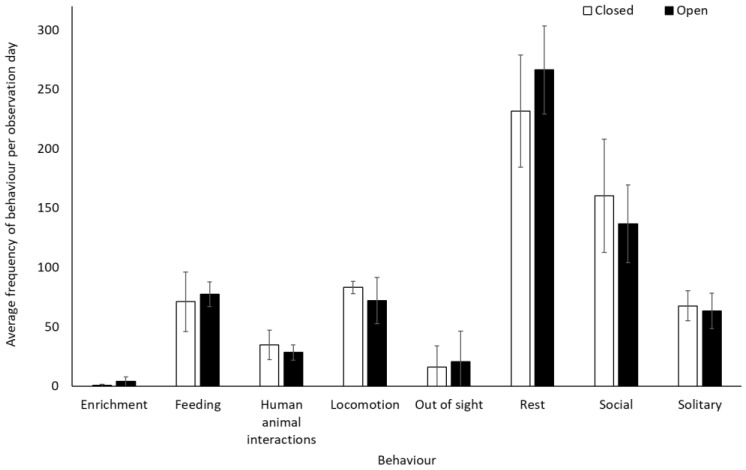
An overview of mean frequency of chimpanzee behaviour during closure and reopening observation periods. Error bars represent standard deviation.

**Figure 4:**
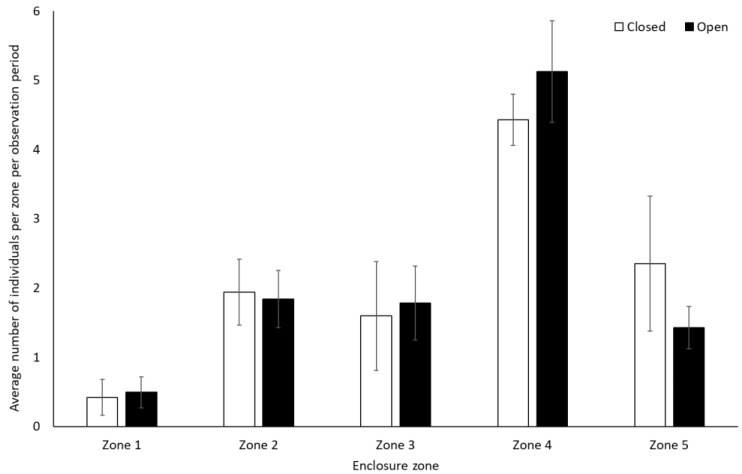
Average number of chimpanzees per enclosure zone per observation period. Error bars represent standard deviation.

Chimpanzees spent more time engaging with enrichment (1.58 ± 0.74, Z = 2.13, *p* = 0.03) and more time feeding (0.25 ± 0.10, Z = 2.37, *p* < 0.02) when the zoo was open to the public than when it was closed. They showed no other statistically significant changes in behaviour between the two conditions (*p* > 0.05, Figure 3, Table S2). They used Zone 4 more frequently when the zoo was open to the public (mean individuals ± SD 5.1 ± 0.7) than when it was closed (4.4 ± 0.4) (U = 3.00, df = 8, Z = −1.98, *p* = 0.05). No other changes were observed in enclosure usage when the zoo was opened or closed to visitors (Zone 1: t(8) = −0.47, *p* = 0.65; Zone 2: t(8) = 0.36, *p* = 0.72; Zone 3: t(8) = 0.44, *p* = 0.67; Zone 5: t(5.317) = −1.850, *p* = 0.12)) (Figure 4). 

**Figure 5:**
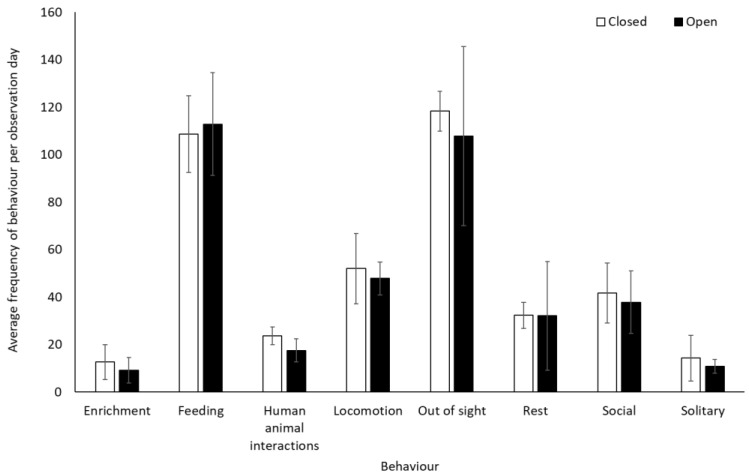
An overview of mean frequency of gorilla behaviour during closure and reopening observation periods. Error bars represent standard deviation.

**Figure 6:**
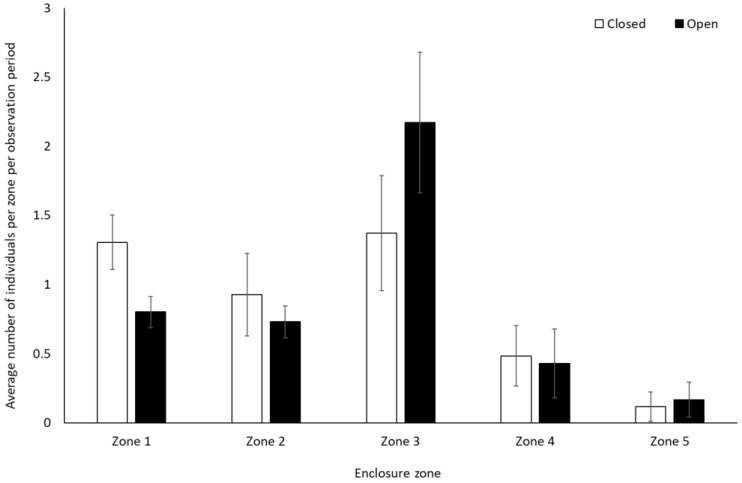
Average number of gorillas per enclosure zone per observation period. Error bars represent standard deviation.

Gorillas engaged in significantly less resting behaviour (−0.28 ± 0.14, Z = 2.01, *p* = 0.04) and spent less time alone (solitary) when the zoo reopened (−0.46 ± 0.20, Z = −2.29, *p* = 0.02) (Figure 5). They also spent significantly less time in Zone 1 (closest to the public) when the site was open to visitors (0.8 ± 0.1) than when it was closed (1.3 ± 0.2) (t(7) = 4.991, *p* = 0.002). There was a trend towards an increased use of Zone 3 when the facility was open (2.2 ± 0.5) compared to when closed (1.4 ± 0.4) (t(7) = −2.337, *p* = 0.05). There was no significant change in the use of Zone 2 (U = 4.00, Z = −1.29, *p* = 0.197), 4 (t(7) = 0.33, *p* = 0.75), and 5 (t(7) = −0.58, *p* = 0.58) (Figure 6).

#### 3.1.2. Physiological Data

There was no significant difference in faecal glucocorticoid metabolites (FGMs) when the zoo was open or closed to the public for any of the study species at Twycross Zoo (bonobo: U = 38.00, df = 21, Z = −1.72, *p* = 0.09; chimpanzee: t(23) = 0.89, *p* = 0.38; gorilla: t(23) = 1.71, *p* = 0.10). Mean ± SD FGMs (pg/mL) when the zoo was closed and open, respectively, were bonobo: 891.6 ± 320.9, 677.8 ± 217.8; chimpanzees: 600.8 ± 265.3, 505.6 ± 262.9; and gorillas: 932.6 ± 237.4, 773.9 ± 208.1. There was no significant variation in FGMs when the zoo was open or closed for any of the study species (bonobo: F = 3.65, *p* = 0.07; chimpanzee: F = 0.02, *p* = 0.89; gorilla: F = 0.18, *p* = 0.67). 

### 3.2. Knowsley Safari 

#### 3.2.1. Frequency of Behaviour

**Figure 7:**
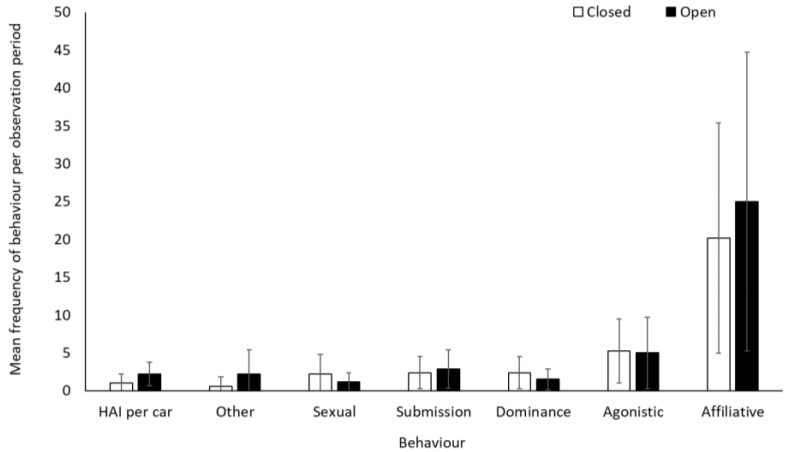
An overview of mean frequency of olive baboon behaviour during closure and reopening observation periods. To account for variation in sample group sizes, data represents an average behaviour frequency per smallest group size (group size 1). Error bars represent standard deviation.

There was no significant difference in frequency of affiliative, agonistic, and submissive behaviours for the olive baboons when the safari park was open or closed to visitors (*p* > 0.05, Table S2). Dominance (−0.5437 ± 0.1972, Z = −2.757, *p* = 0.006) and sexual behaviour (−0.6980 ± 0.2349, Z = −2.971, *p* = 0.003) were lower when the safari park was open than when closed. Human–animal interactions (number of vehicle contacts) were more frequent when open (exposed to visitors’ cars and the ranger’s vehicle) than when closed (exposed only to the ranger’s vehicle) (1.824 ± 0.201, Z = 9.093, *p* < 0.001), as were “other” behaviours (1.4436 ± 0.4551, Z = 3.172, *p* = 0.002) (Figure 7). Average rates (mean ± SD) of human–animal interactions per car were 4.6 ± 5.7 and 6.9 ± 3.9 when the facility was closed and open, respectively.

There was a negative relationship between performance of sexual behaviour and number of cars at the baboon enclosure when the safari park was open to the public (−0.2806 ± 0.1016, Z = −2.761, *p* = 0.01), indicating that sexual behaviours were being performed less frequently when there were more cars in the baboon enclosure. There was no relationship between the number of cars in the enclosure and any other of the other behaviours (*p* > 0.05; Table S3).

#### 3.2.2. Physiological Data

There was no significant change in FGMs for the baboons when the safari park was open or closed to the public (U = 236.00, df = 50, Z = −0.09, *p* = 0.93). Mean ± SD FGMs (pg/mL) for closed and open periods, respectively, were 1582.4 ± 400.1, 1636.6 ± 595.6. There was no significant variation in FGMs when the safari park was open and when it was closed (F = 0.85, *p* = 0.36). 

## 4. Discussion

Previous “visitor effects” research has predominantly focused on the impacts of differing numbers of zoo visitors on zoo-housed primates, and has highlighted the complexity of the relationship between zoo visitors and zoo-housed non-human primates [13,18,43]. The aim of this research was to investigate the impact of facility closures (resulting in no visitors present) and subsequent reopenings to zoo visitors on behavioural and physiological parameters of welfare in a selection of primate species (bonobos, chimpanzees, and western lowland gorillas) housed within a zoo environment, and olive baboons housed in a safari park. Using periods when the facilities were closed due to the COVID-19 global pandemic allowed an enhanced understanding of whether and how animals were altering their behaviour or had changes in faecal glucocorticoid metabolite (FGM) levels when there were no visitors present, and how this compared to when visitors returned to zoos. This research highlighted some short-term behavioural changes but no changes in FGMs during either the closures or the subsequent facility reopenings. 

### 4.1. Behavioural Changes

When the zoo was open to the public, bonobos and gorillas spent less time alone and gorillas engaged in less resting behaviour, which is indicative of a potentially negative impact from zoo visitors. The reduction in resting behaviour has been previously observed in other gorilla groups and suggests primates are disrupted by visitors [44]. This is similar to what has been observed in other primate species, including Diana monkeys (*Cercopithecus diana*) [45] and mandrills (*Mandrillus sphinx*) [46]. However, chimpanzees showed increased activity, spending longer engaging in feeding and interaction with enrichment. Similar findings were also reported in Diana monkeys, who showed an increase in feeding behaviour in response to visitor numbers [45]. Whilst this is indicative of increased activity, which is consistent with other studies, the increase in feeding and enrichment-related activity is opposite to what other studies report (e.g., [47]), and highlights the variation in responses of primates to zoo visitors. The increase in feeding activity and the absence of heightened interest/interaction with humans suggests that the presence of visitors may have been stimulating for the chimpanzee group, but visitors were not causing extreme negative responses or overt interest.

Positive social interactions are an indicator of positive welfare for zoo-housed species [48], and social activity is typically reported to reduce in primate species in relation to increased visitor numbers [17,19,46,49]. The results of this study indicated the opposite for both the bonobos and gorillas. Similar findings have been reported in ebony langurs (*Trachypithecus auratus*) [50], white-crowned mangabeys (*Cercocebus torquatus*) [51], and chimpanzees [52]. Stable and appropriate social groups within zoos have been identified as a positive influence on zoo animal welfare, with affiliative (or positive) social interactions buffering against stress [53]. It has been suggested that increased affiliative behaviours in the presence of visitors can provide reassurance in primates, with these behaviours being used to alleviate visitor-induced stress [50,51]. It is not clear whether the presence of visitors within the zoo were a causal factor in the increase of social interactions. However, the increase of positive social interactions, without an increase in aggressive behaviour, suggests that even if the animals were increasing sociality in order to buffer stress, they were able to behaviourally respond to the new situation (visitors back in zoos) without experiencing reduced welfare. Similar increases in sociality were also recorded in meerkats, another social species, when facilities reopened to the public [24]. This shows the potential adaptability of zoo species to ever-changing environments. 

Whilst the reduced resting behaviour in gorillas when the zoo was open to the public may be indicative of increased restlessness, or temporary stress caused by the return of visitors to the zoo, it is also possible that the visitors returning to the zoo were a positive stimulant for the gorillas. This difference in activity between gorillas and the other primates may reflect a more sedentary species. On average, gorilla mass is heavier than chimpanzees and in the wild their mean daily walking distance is lower, whilst their total energy expenditure per day is higher [54]. Gorillas may therefore have rested more during closure periods when there was no stimulation from zoo visitors. Anecdotal reports from zoological facilities during COVID-19 lockdown periods highlighted the fact that primates were looking out for keepers [55], and so these individuals could have been responding to the increased stimulation when visitors returned. It is, however, important to bear in mind that the time frame over which these data were captured was relatively short, so it is possible that the gorillas, as has been reported in other species [26,56], were taking longer than the bonobos or chimpanzees to rehabituate to zoo visitors when facilities reopened.

Olive baboons also displayed some behavioural changes, with increased human–animal interactions (number of vehicle contacts during the observation) and decreased performance of sexual behaviours when the safari park was open. In wild baboons, mating patterns are varied, but generally, sexual behaviours only occur in the initial stages of a sexual swelling or during maximum tumescence indicative of ovulation [57]. Females produce acoustic and olfactory signals when fertile, and therefore sexual occurrences rarely occur outside of ovulation [58,59]. Due to the size of the baboon troop at Knowsley Safari it is estimated that there are usually approximately 20 females in oestrus at any one time, with higher-ranking females becoming pregnant immediately post-nursing, rather than individuals falling into specific breeding cycles as is more commonly observed in the wild. Anecdotally, keepers attributed the increased sexual behaviour to an increase in imitation behaviour being performed by the juvenile members of the troop when they did not have the alternative stimulation from the presence of moving vehicles in their enclosure (Davies Walsh & Armstrong, pers comm). 

### 4.2. Enclosure Use: Impacts of Enclosure Design and Visitor Numbers

The bonobos did not significantly change their enclosure usage between periods of time when the zoo was closed and open to the public. The chimpanzees used Zone 4 more when the zoo was opened than when closed. The gorillas used the zone of their enclosure which was nearest to the public significantly less when the zoo was open to the public and showed a tendency towards using the middle of their enclosure more. Other research has highlighted the lack of change in enclosure use in gorillas in areas nearest to visitors [22]. However, changes to enclosure use in response to increased visitor density or visitor noise have been reported in a number of other primate species, including lion-tailed macaques [19], gorillas [20], white-cheeked gibbons, and siamangs (*Hylobates syndactylus*) [21]. As has been reported in Kuhar [60], it is possible that the change in space use recorded in the gorillas in this study was an avoidance mechanism when visitors returned. Indeed, the closure periods may have reduced the tolerance of the gorillas to visitor presence. Avoidance behaviours have been known to increase in response to increased visitor number and noise levels at enclosures for orangutans [61], and a number of studies have reported gorillas turning their back on enclosure windows (another behaviour indicative of public avoidance) in relation to increased visitor presence [62,63]. Strategic use of enclosures to avoid human–animal interactions has also been reported in animals in petting zoos [63], and changes in enclosure usage/visibility of animals following periods of closure during the COVID-19 pandemic have also been reported in red kangaroos [26], Grevy’s zebra (*Equus grevyi*) [56], and tokay geckos (*Gekko gecko*) [64]. The short-term behavioural modification in terms of altered space use may therefore represent a coping mechanism that the gorillas in this study employed when the zoo was open to the public.

Great apes are known to use a relatively limited area of their available space, with both chimpanzees and gorillas being highly selective in their space use [65]. Space use is an important consideration when investigating the impact of zoo visitors on animal experiences, especially if external stimuli (e.g., zoo visitors) impact on an animal’s ability to access and utilise biologically relevant resources [11]. Provision of opportunities to enable primates to avoid visitors or enclosure modifications which have provided opportunities for reduced visual contact with visitors has led to behavioural indicators of improved welfare, reduced aggression, vigilance, and abnormal repetitive behaviours in gorillas [47,66], and reduced aggression in black-capped capuchin monkeys (*Cebus apella*) [67]. Providing animals with the opportunity to retreat from visitors may thus prevent the onset of negative behavioural responses to visitors. Traditional zoo and safari park exhibits as discussed in this research represent very different environments in terms of animal–visitor interaction opportunities. In more traditional zoo enclosures, animals are viewed by visitors through protective windows or mesh. Modern zoo exhibits will incorporate naturalistic features or opportunities for animals to move away from the visitors but it is possible that this is linked to displacement rather than choice. Drive-through safari park enclosures allow the animals the choice to engage with the visitors by moving towards, or in the case of baboons, on top of, the vehicles or not. With more opportunity for choice, it is possible that this will increase positive welfare outcomes for individuals in these environments [68]. When a group of orangutans were offered the opportunity to choose whether to face the public viewing area or not, the studied animals showed a preference for positioning themselves where they could see the public viewing area [69]. 

When the safari park drive-through was open to visitors (compared to closed), baboons increased contact with cars in the exhibit (as compared to contact made with the ranger’s vehicle). The type of interactions with vehicles was not recorded during data collection and thus the valence of this engagement is unknown. However, baboons made an active choice to approach vehicles, rather than ignore or avoid them. Baboons are highly intelligent and are renowned for active engagement with cars going through the drive-through at Knowsley Safari [70]. However, once the safari park had reopened to visitors, this behaviour did not continue to increase in relation to the increased number of cars in the enclosure. This suggests that there may be a saturation point at which up until that point, the number of visitors/vehicles are a stimulus for the baboons, and after that threshold, they do not continue to exponentially stimulate the baboons. The managed opening of the safari park and the requirement for pre-booked tickets post-COVID-19 closures led to enhanced visitor control in this and other collections. This led to maximum numbers of people on site at any one time and, to some degree, limited cars using the drive-through. Visitor behaviour was not recorded during this study and so it is not possible to say whether cars used the drive-through in the way they normally would, or whether due to the reduced number of vehicles traffic was more “free flowing” than stationary. This management may have contributed to the “cap” on how stimulating the cars in the enclosure were. Expanding this research to consider behavioural responses of animals in drive-through enclosures in terms of frequency, type, and valence of interactions with visitor vehicles when sites resume “normal” operations would enhance our understanding of this effect.

### 4.3. Physiological Data

There were no significant differences in FGMs between open and closed periods for any of the study species, nor was there any greater variance in FGM levels in the samples between open or closed periods. The absence of significant changes in the FGMs of the study groups suggest there were no extreme negative implications for the study animals, either during closures or subsequent reopening periods. These findings are not necessarily surprising, as other authors have found contrasting evidence on the effect of visitors on faecal stress hormone levels in captive primates. Some studies have indicated that zoo-housed primates are negatively affected by large numbers of noisy zoo visitors, with a positive correlation between cortisol levels and visitor numbers [71], and others suggest that zoo visitors can be beneficial to captive primates, with visitor presence considered as enrichment [72]. It was beyond the scope of this opportunistic study to identify individual faecal samples and thus FGM data are from pooled samples. The hypothalamic–pituitary–adrenal (HPA) system, which stimulates adrenal release of cortisol, allows organisms to adapt to physical and psychosocial changes in their environments [73]. However, individuals perceive and respond differently to stressors, with no two individuals experiencing the same environment in the same way [74], and there is evidence that HPA activity may differ between individuals and situations [73]. It is therefore possible that subtle, individual-level differences in FGMs were being masked by others in the group. However, there were no overt behavioural changes indicative of reduced welfare or excessive negative stress. Furthermore, the lack of significant differences in variability between samples collected when facilities were open and when facilities were closed suggests there was not a significant amount of variation during the two data collection periods physiologically across the study groups.

### 4.4. Study Limitations 

The limitations highlighted here were taken into consideration by the authors during the interpretation of the findings. Despite the shortcomings, which are inherent in data collected during such unique circumstances, this research adds a new dimension to our understanding of the impact of zoo visitors on animals. The authors believed it was prudent to highlight the limitations of this work to enable readers to consider them in their own interpretations of the paper. 

The sampling methods employed were relatively basic, in order to allow this research to be simple, quick, and repeatable across enclosures. A loss of detailed information occurred at the individual level but behavioural and physiological data were reliably captured at the group level. Data on enclosure usage were collected using a basic method of approximate proximity to visitor viewing areas (closest fifth through to furthest away fifth). This did not consider biological relevance nor size of zone in relation to the visitor viewing area. Whilst simplistic, this did allow us to capture data reliably and consistently on whether the animals had changed their proximity to the visitor viewing area during site closures and subsequent reopening periods. Due to the limitations of the data collection protocol, it was also decided to use relatively simple modelling methods (negative binomial GLMs) to analyse the data. The use of scans from within the same day technically led to a lack of independence within the dataset; however, owing to a small sample size, it was decided that generalised linear mixed models with day as a random factor would have led to overfitted models and thus were not appropriate for this dataset. 

Previous research has highlighted the impact of visitor number and visitor behaviour on how animals perceive the interaction, particularly in regards to the interactions between zoo primates and visitors [61,75,76]. During this study it was not possible to capture the number of visitors at the enclosures at Twycross Zoo, nor the visitor dwell time or visitor behaviour. Many facilities were implementing pre-booking systems (with a cap on visitor numbers), one-way systems, and social distancing requirements to ensure visitor safety, in line with guidance from The Global Association of Attractions Industry and the British and Irish Association of Zoos and Aquariums (BIAZA) [77,78]. This was also the case at Twycross Zoo (Rendle, pers comm). The requirements for only known persons to be looking into an enclosure together may have reduced the large groups of crowds which can build up at zoo enclosures. Furthermore, no keeper talks were being undertaken during these observation periods, which may have prevented the increased visitor number and subsequent impacts on animals normally associated with these events [13]. Whilst the impact of visitor presence/behaviour during observations during “open” periods at Twycross Zoo could not be captured, it is believed that the restrictions in place during the observation periods minimized excessively large crowds or antisocial behaviour. At Knowsley Safari the number of visitors was based on the mean number of cars travelling through the drive-through enclosure during the observations and is thus a measure of the “traffic”. Again, this was controlled due to the phased reopening and thus gave an opportunity to look at the impact of visitors returning to the safari park on baboon behaviour. 

Another point to bear in mind is that no information was captured in relation to weather conditions, which has also been known to impact primate behaviour within zoos [79]. Whilst weather conditions on site may have impacted on animal behaviour, data were collected at a similar time of year and so the likelihood of extreme variation in weather conditions across the species is likely to be minimal. As not all species showed significant behavioural change, it is likely that the results are indicative of behavioural changes related to the presence/absence of the public, rather than a reaction to weather events.

Finally, as has previously been highlighted, a lack of ability to identify individual faecal samples led to a necessity to analyse FGMs at a group level. Hormonal differences naturally occur among different individuals, depending on age, sex [80], and rank [81] of the subjects, which could thus have impacted on the overall FGM levels. This prevented understanding of the impacts of reopening on individuals. However, as has been highlighted in relation to visitors, physiological responses can be variable [74]. The lack of physiological responses recorded here may thus be a function of the methodological approach. The absence of significant negative behavioural indicators supports the absence of physiological changes; however, this is an area that would need more detailed investigation at an individual level to make more robust conclusions in relation to physiological impacts of the closures on the studied animals.

### 4.5. Directions for Future Research

As has been previously highlighted, to the authors’ knowledge, no published research has investigated the impact of the changed animal–visitor dynamic which may be present within a safari park environment as compared to a zoo. This research suggested that the presence of cars in the safari park environment was a stimulant for the olive baboons. However, it is unknown whether this positive stimulation from visitors extends to other species. Furthermore, due to the lack of comparison with a zoo-housed group of olive baboons, it is not possible to say whether this was an effect of species difference, group size, or type of housing. We advocate that future research looks at the differences between zoos and safari parks in relation to the impact of zoo visitors on animals, undertaking cross-species comparisons where possible. Special consideration should be given to the potential welfare implications for species who move from one type of facility to the other (e.g., from safari parks to zoos or vice versa), to determine whether the change in the method by which visitors are presented has implications for welfare of animals. 

Finally, research has shown that past experience shapes future lives of zoo animals [10,18] and can influence how they perceive their environments, with individuals within zoos experiencing environments differently [82]. There are a number of individual-level differences which could impact the effect of visitors on animals. Previous research into the differences between high and low visitor numbers has suggested that individual animal responses to visitors may be affected by animal personality, age of individuals, historical interactions with people, or individual rearing history (e.g., captive or parent-reared) [14,21,60,83]. If animals have experienced previous negative situations with visitors, then there is the potential for this to shape future interactions. Investigation of the impact of individual differences on the study animals was beyond the scope of this research. We advocate that future work should seek to understand, where possible, the impact of individual-level differences on animal experiences in relation to HAIs in order to advance understanding and support evidence-based management. 

## 5. Conclusions

The results of this work, in line with other “visitor effects” literature and research into the impacts of the COVID-19 facility closures on zoo animals, highlights the variable impacts of the closures on the primates studied. Although it is not possible to accurately state whether the impacts of visitors were positive, negative, or neutral, the behavioural changes observed in the baboons without exponential responses to increased visitors suggests that visitors may have been a stimulant but that there was a threshold after which they were not increasingly stimulated by the visitors. Changes in chimpanzee activity likewise suggested that visitors were a stimulant, whereas the altered enclosure use in gorillas and reduced periods of time spent in solitary by gorillas and bonobos suggests that these species may have been altering activity to reduce the potential overstimulation or stressors experienced during the reopening. The opportunity for choice enabled these species to modify their behaviour, and the absence of overt indicators of reduced welfare suggests these species were managing their own experiences in an effective manner. Behavioural changes and observed changes in enclosure use highlights the adaptability of zoo species to their environments, including the presence of zoo visitors. We recommend that future work should seek to understand the impact of individual-level differences in relation to the effects of zoo visitors and that differences between animal experiences in zoos and safari parks are further explored in a range of species. 

## Figures and Tables

**Table 1:** Details of study sites and periods of data collection (M = male, F = female).

Study Site	Species (Number of Individuals)	Period of Data Collection	Approximate Percentage of Enclosure Perimeter Accessible by Visitors via Viewing Windows	Number of Separate Observation Periods		Total Number of Observation Scans (Hours of Observations)	
				Closed	Open	Closed	Open
Twycross Zoo	Bonobo (4 M, 8 F)	October–November 2020	68.4%	6	4	288 (23.6 h)	394 (32.3 h)
	Chimpanzee (4 M, 7 F)	November–December 2020	33.3%	5	5	302 (24.8 h)	306 (25.1 h)
	Western lowland gorilla (3 M, 3 F)	November 2020–January 2021	45%	3	6	202 (16.6 h)	376 (30.8 h)
Knowsley Safari	Olive baboon (192)	April–September 2020	N/A (Drive-through enclosure)	45	48	1350 (22.5 h)	1440 (24 h)

**Table 2:** Simplified ethogram which was used for data collection and analysis for bonobos, chimpanzees, and western lowland gorilla (adapted from Leeds et al. [31], Leeds et al. [32], and Gartner and Weiss [33]).

Behaviour	Description
Locomotion	The animal is moving in any direction on any number of limbs without picking up food.
Resting	The animal is either sitting, standing, or lying down and is not doing anything else. They may also be sleeping, where their eyes are closed. They will not move from the immediate area.
Feeding	Animal moves slowly around picking up small pieces of food. Animal consumes food item in mouth. Chewing may occur where mouth moves whilst item is in mouth before swallowing. Small items may not be chewed but swallowed as soon as item is placed in mouth.
Social	Animal is interacting with another animal directly, can be tactile, visual, or auditory contact between two or more animals within the vicinity.
Enrichment	Animal is interacting with an item placed in the enclosure by a human specifically for the use of enrichment. This must be direct interaction.
Solitary	Animal is performing an “active” activity away from other animals.
Human	Animal is interacting or watching a human.
Out of Sight	Animal is not visible to the observer.

**Table 3:** Simplified ethogram (based on Molesti et al. [34]) which was used for data analysis of olive baboons.

Behaviour	Description
Affiliative	Behaviours deemed positive, those strengthening or maintaining ties between conspecifics/relatives; Lip Smack, Touch, Sniff, Embrace, Groom, Mutual Groom, Social Play, Play Facial Expression.
Agonistic	Behaviours deemed negative, those showing aggression, often with gains to the aggressor and at the detriment of the receiver; Threat, Head Bob, Bared Teeth, Display, Ground Slapping, Chase, Lunge, Contact Aggression, Fight.
Dominance	Behaviours establishing or maintaining a hierarchy within the troop. The initiator is therefore of a higher status than the receiver; Disperse, Stare, Steal, Fight over mate, Infant Aggression.
Submission	Behaviours establishing or maintaining a hierarchy within the troop. The initiator is therefore of a lower status than the receiver; Fear Grin, Social Present, Lower Body Position, Avoid, Flee.
Sexual	Behaviours functioning for acquiring a mate and/or reproduction.
Human interaction	Any interaction with a human including approaching and interacting with a visitor’s or keeper’s vehicle.
Other	Any other social behaviour not otherwise described, including easily audible vocalisations.

**Table 4:** An overview of number of faecal samples collected per study species at the two data collection sites.

Study Site	Period of Faecal Sample Collection	Species	Number of Faecal Samples	
			Closed	Open
Twycross Zoo	October–November 2020	Bonobo	11	12
		Chimpanzee	11	14
		Gorilla	12	13
Knowsley Safari	July–November 2020	Baboon	13	39

**Table 5:** Group size categories for the olive baboons.

Group Size Category	Number of Individuals
1	1–10
2	11–20
3	21–30
4	31–40
5	41–50
6	51+

**Table 6:** An overview of behaviours recorded per species and inferential statistics performed.

Species	Behaviours on Which Negative Binomial Regression Models Were Performed	Enclosure Usage
Bonobo	Locomotion, resting, feeding, engaging with enrichment, solitary, human interactions, and out of sight.	Zone 1: t-test for independent samples Zone 2–4: Mann–Whitney U-test Zone 5: Mann–Whitney U-test using transformed data (ln(x + 1^−10^))
Chimpanzee		Zone 1–3: t-test for independent samples Zone 4: Mann–Whitney U-test Zone 5: t-test for independent samples using transformed data (ln(x))
Gorilla		Zone 1, 3–5: t-test for independent samples Zone 2: Mann–Whitney U-test using transformed data (ln(x))
Olive baboon	Dominance, sexual, human–animal interactions, affiliative, agonistic, submission, and other.	

## Data Availability

Data available from the corresponding author upon reasonable request.

## Supplementary Materials

The following supporting information can be downloaded at: https://www.mdpi.com/article/10.3390/ani12131622/s1, Table S1: Extended ethogram used to collect data during observations of olive baboons at Knowsley Safari; Table S2: Model outputs for number of observations of behaviours during the zoo closure and reopening periods; Table S3: Model outputs for number of observations of behaviours being performed by the Olive baboons in relation to number of cars in the enclosure.
