# Supplementary material for: The Impact of COVID-19 Zoo Closures on Behavioural and Physiological Parameters of Welfare in Primates
Source: Animals (Basel). 2022 Jun 24;12(13):1622. doi: 10.3390/ani12131622 (PMC9265073; doi:10.3390/ani12131622)
Supplement: Supplementary file 1 [file animals-12-01622-s001.zip › animals-1750171-supplementary/Williams et al_Supplementary material/Williams et al_Table S1.pdf]

Table S1. Extended ethogram used to collect data during observations of olive baboons at Knowsley Safari.

**Behaviour**	**Definition**
Lip Smack	Rapid, repetitive opening and closing of the lips, directed at a conspecific
Touch	Briefly touch conspecific
Sniff	Sniff a conspecific
Embrace	Both arms wrap around or greet conspecific
Groom	Moving the fur of a conspecific, searching for and removing foreign objects
Mutual Groom	More than two individuals groom each other at the same time
Social Play	Play with a conspecific
Play Facial Expression	Open mouth gesture, no teeth bared
Threat	Staring at a conspecific with raised eyebrows
Head bob	Moving the head up and down aimed at a conspecific
Bared teeth	Large 'yawn' displaying canines, aimed at a conspecific
Display	Shaking of inanimate object, i.e. Mesh/trees etc
Ground Slapping	Hitting the ground around them, aimed at a conspecific
Chase	Individual runs after a conspecific for at least two metres, not affiliative play
Lunge	Individual moves upper body and/or throws body towards a conspecific in a threatening manner
Contact Aggression	Individual bites, pulls or grabs a conspecific, regardless of if other responds
Fight	Two or more individuals make aggressive contact for more than 3 seconds
Fear Grin	Upper and lower lips draw back showing all teeth, individual looks around
Social Present	Individual orients rear end towards a conspecific, in a non-sexual context
Lower Body Position	Individual crouches/hides with torso low to ground
Avoid	Individual moves away after making eye contact with a more dominant conspecific, or when a dominant individual approaches
Flee	Run away at least two metres in response to aggression

Disperse	When individual approaches, other conspecifics avoid/move from area at least two metres
Stare	Individual makes eye contact with a conspecific whilst raising eyebrows to reveal white part of eye, conspecific breaks eye contact/moves
Steal	Individual takes an item of value from another conspecific ie. Car part, food, enrichment etc.
Fight over mate	2+ individuals make contact aggression over a female
Infant Aggression	An unrelated adult male, takes and is aggressive towards an infant belonging to a female within another males' harem
Sexual	Any behaviour related to reproduction
Investigate	An individual sniffs/touches the perio-anal area of another conspecific
Other	Any other behaviour not defined
Human Interaction	Any interaction with a human, visitor/keeper
