# Supplementary material for: The Impact of COVID-19 Zoo Closures on Behavioural and Physiological Parameters of Welfare in Primates
Source: Animals (Basel). 2022 Jun 24;12(13):1622. doi: 10.3390/ani12131622 (PMC9265073; doi:10.3390/ani12131622)
Supplement: Supplementary file 1 [file animals-12-01622-s001.zip › animals-1750171-supplementary/Williams et al_Supplementary material/Williams et al_Table S2.pdf]

Table S2. Model outputs for number of observations of behaviours during the zoo closure and reopening periods

Species/behaviour	Model estimate (β_1) ±SE	Z value	Significance
**Bonobo**			
Locomotion	-0.05±0.05	-0.94	p=0.35
Rest	0.17±0.18	0.91	p=0.36
Feeding	-0.03±0.13	-0.24	p=0.81
Social	-0.08±0.19	-0.43	p=0.67
Solitary	-0.31±0.11	-2.88	p=0.004
Enrichment	-0.59±0.53	-1.10	p=0.27
Human-animal interaction	0.22±0.52	0.42	p=0.68
Out of sight	0.29±0.20	1.46	p=0.14
**Chimpanzee**			
Locomotion	-0.16±0.10	-1.57	p=0.12
Rest	0.13±0.08	1.59	p=0.11
Feeding	0.25±0.10	2.37	p=0.02
Social	-0.16±0.13	-1.33	p=0.12
Solitary	-0.08±0.13	-0.62	p=0.53
Enrichment	1.59±0.74	2.13	p=0.03
Human-animal interaction	-0.22±0.19	-1.14	p=0.26
Out of sight	0.22±0.73	0.31	p=0.76
**Western lowland gorilla**			
Locomotion	-0.009±0.12	-0.07	p=0.94
Rest	-0.28±0.14	2.01	p=0.04
Feeding	0.11±0.10	1.13	p=0.26
Social	-0.005±0.22	-0.02	p=0.98
Solitary	-0.46±0.20	-2.29	p=0.02
Enrichment	-0.25±0.32	-0.80	p=0.43
Human-animal interaction	-0.23±0.15	-1.50	p=0.13
Out of sight	-0.4±0.19	-0.20	p=0.84
**Olive baboons**			
Affiliative	0.19±0.14	1.39	p=0.17
Agonistic	-0.06±0.16	-0.38	p=0.70
Submission	0.14±0.19	0.73	p=0.47
Dominance	-0.54±0.20	-2.76	p=0.006
Sexual	-0.70±0.23	-2.97	p=0.003
Human-animal interactions	1.82±0.20	9.10	p<0.001
Other	1.44±0.46	3.17	p=0.002
