# Supplementary material for: The Impact of COVID-19 Zoo Closures on Behavioural and Physiological Parameters of Welfare in Primates
Source: Animals (Basel). 2022 Jun 24;12(13):1622. doi: 10.3390/ani12131622 (PMC9265073; doi:10.3390/ani12131622)
Supplement: Supplementary file 1 [file animals-12-01622-s001.zip › animals-1750171-supplementary/Williams et al_Supplementary material/Williams et al_Table S3.pdf]

Table S3. Model outputs for number of observations of behaviours being performed by the Olive baboons in relation to number of cars in the enclosure

| Species/behaviour | Model estimate ($\beta_1$) ±SE | Z value | Significance |
|---|---|---|---|
| Affiliative | -0.08±0.06 | -1.34 | p=0.18 |
| Agonistic | -0.08±0.08 | -0.99 | p=0.32 |
| Submission | -0.12±0.08 | -1.39 | p=0.16 |
| Dominance | -0.06±0.09 | -0.71 | p=0.48 |
| Sexual | -0.28±0.10 | -2.76 | p=0.01 |
| Human-animal interactions | 0.08±0.07 | 1.16 | p=0.25 |
| Other | 0.03±0.19 | 0.19 | p=0.85 |
